# A Scoping Review of Recurrent Post‐Tonsillectomy Hemorrhage in Children

**DOI:** 10.1002/lary.70304

**Published:** 2025-12-08

**Authors:** Kathleen R. Billings, Saied Ghadersohi, Andrea J. Fawcett, Inbal Hazkani

**Affiliations:** ^1^ Division of Pediatric Otolaryngology‐Head and Neck Surgery Ann & Robert H. Lurie Children's Hospital of Chicago Chicago Illinois USA; ^2^ Department of Otolaryngology‐Head and Neck Surgery Northwestern University Feinberg School of Medicine Chicago Illinois USA; ^3^ Department of Clinical & Organizational Development Ann & Robert H. Lurie Children's Hospital of Chicago Chicago Illinois USA

**Keywords:** pediatric patients, post‐operative bleeding, post‐tonsillectomy hemorrhage, tonsillectomy

## Abstract

**Objective:**

Tonsillectomy, with or without adenoidectomy, is the most common major surgical procedure performed in children. The risk and incidence of primary and secondary post‐tonsillectomy hemorrhage (PTH) have been well described. The goal of this scoping review is to analyze the incidence and risk factors for recurrent PTH, and to map existing evidence to identify knowledge gaps.

**Data Sources:**

CINAHL, Cochrane Library, Embase, Google Scholar, and Ovid Medline.

**Review Methods:**

The study followed PRISMA‐ScR guidelines. Three reviewers independently screened studies, including those reporting the incidence of PTH and recurrent PTH in children.

**Results:**

There were 229 recurrent PTH episodes described in the 11 manuscripts included in this analysis, with 7.1% of initial PTH cases re‐bleeding (0.33% of total tonsillectomy cases). Management of recurrent PTH involved surgical intervention and observation. Oropharyngeal findings at the time of the initial PTH and management strategy for the initial PTH were not associated with increased recurrence rates. The indication for tonsillectomy, NSAID usage, and surgical technique were not associated with recurrent PTH when reported. Across studies, there was wide heterogeneity in how recurrent bleeding was defined, and inconsistent reporting of timing and outcomes. The predictive value of laboratory screening for occult coagulopathies in children with multiple bleeds was unclear.

**Conclusions:**

No clear risk factors for recurrent PTH were identified from the pooled analysis. This scoping review highlights major research gaps, including the need for standardized definitions and severity grading, prospective multicenter data to clarify predictors of recurrence, and systematic evaluation of hematologic screening protocols.

## Graphical abstract

The goal of this scoping review was to analyze the incidence and risk factors for recurrent peritonsillar hemorrhage (PTH), and to map existing evidence to identify knowledge gaps. CINAHL, Cochrane Library, Embase, Google Scholar, and Ovid Medline were searched. Eleven manuscripts were included in this analysis, with 7.1% of initial PTH cases re‐bleeding. No clear risk factors for recurrent PTH were identified from the pooled analysis. This scoping review highlights major research gaps, including the need for standardized definitions and severity grading, prospective multicenter data to clarify predictors of recurrence, and systematic evaluation of hematologic screening protocols.

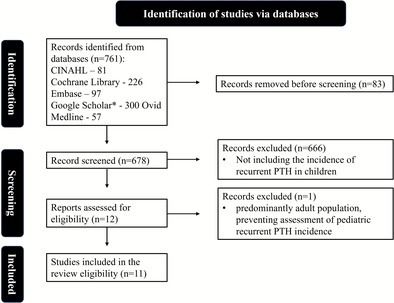

## 1 Introduction

Tonsillectomy, with or without adenoidectomy (T&A), is the most common major surgical procedure performed in the United States, with estimates of up to > 500,000 done yearly in children < 15 years of age [1, 2]. Post‐tonsillectomy hemorrhage (PTH) is the most common complication after T&A surgery, and estimates of frequency are wide ranging: 0.1%–5.8% for primary bleeding (within 24 h of surgery), and 0.2%–7.5% for secondary bleeding (after 24 h) [2]. The Clinical Practice Guideline for tonsillectomy in children recommends that clinicians follow up with their patients post‐operatively and document their PTH rates annually. If rates are higher than reported norms, this would give the clinician the opportunity to “reassess their process of care and improve quality” [1].

A number of studies have analyzed the risk of initial PTH based on technique and post‐operative medications, for example, ibuprofen [3, 4, 5, 6, 7, 8]. In more recent years, with the increased usage of tranexamic acid (TXA) in Emergency Departments (ED) for the management of minor PTH cases, the management strategies for those with PTH have continued to evolve [9, 10]. Less often studied is the rate of and risk factors for *recurrent* PTH, defined as two or more episodes of bleeding after tonsillectomy. The true rate of 2 or more bleeds may be underappreciated as minor bleeding instances may be underreported by parents and caregivers. Several studies have analyzed additional bleeding events in patients hospitalized for observation after an initial PTH and have questioned whether patients reporting to the ED for PTH should be admitted for monitoring of additional bleeding episodes [11, 12, 13].

Given the uncertainty of the incidence, risk factors, and management suggestions for those with recurrent PTH, a scoping review of the available literature was performed to analyze these variables. The literature related to recurrent PTH is primarily case reports and series, limiting the understanding of the extent of the problem, and this drove the decision to perform this review. The goals of this review are to provide insight into the incidence and significance of this occurrence, summarize known risk factors and management strategies, and identify critical gaps in definitions, diagnostic strategies, and outcomes reporting. The knowledge gained from this review may be used to effectively counsel patients and their families about expectations for additional bleeding episodes after an initial PTH, and to highlight priorities for future prospective research.

## 2 Methods

### 2.1 Search Strategy

**FIGURE 1:**
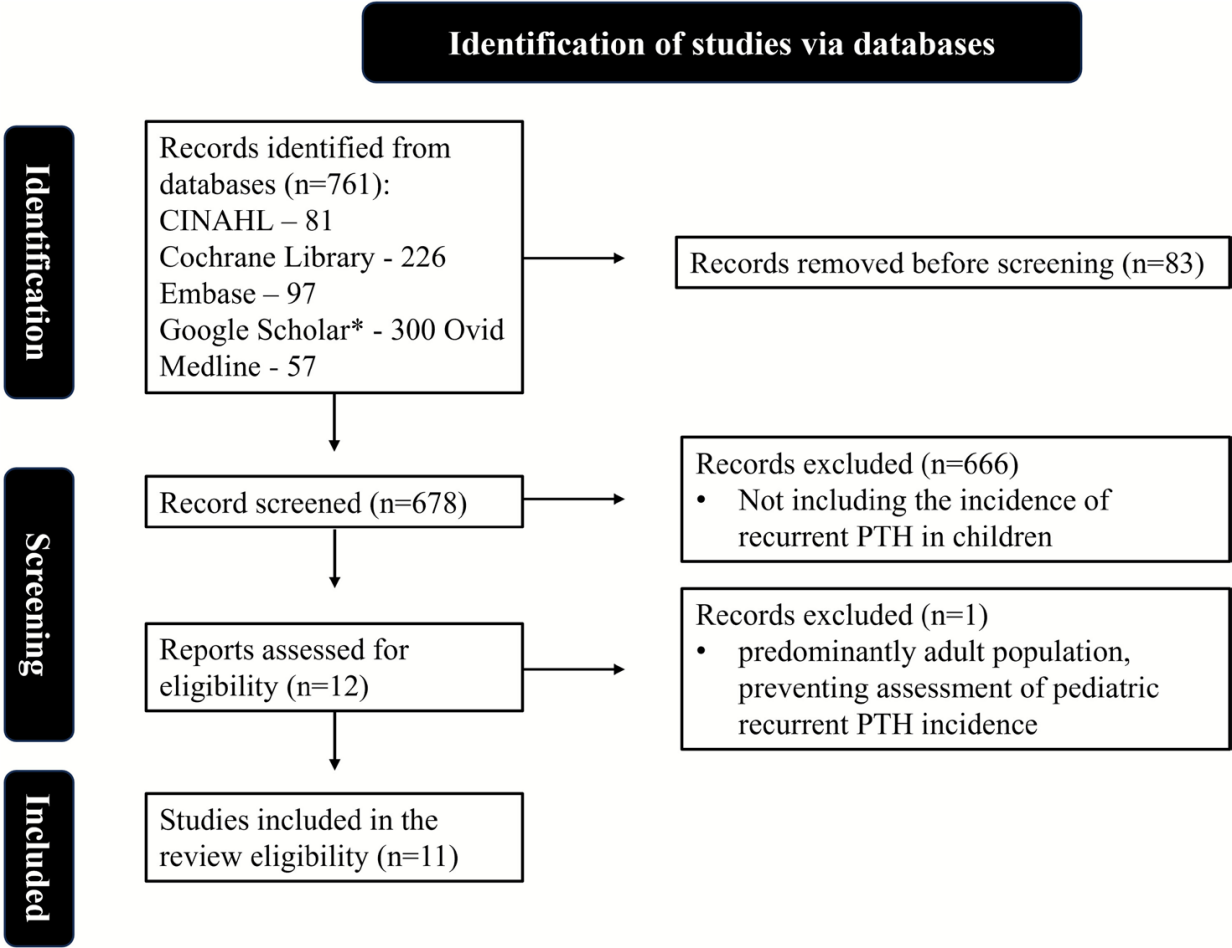
Preferred Reporting Items for Systematic Reviews and Meta‐Analyses (PRISMA) flow chart. (Only the first 200 records were exported).[Color figure can be viewed in the online issue, which is available at www.laryngoscope.com]

As this is a scoping review of the existing literature, the analysis was deemed exempt from IRB approval. A comprehensive search strategy was designed by a medical librarian (AJF) using keywords and controlled vocabulary terms related to pediatric postoperative tonsillectomy patients, bleeding/hemorrhage, and cautery. This review used the JBI Methodology for Scoping Reviews [14] and adhered to the Preferred Reporting Items for Systematic Review and Meta‐Analysis Extension for Scoping Reviews (PRISMA‐ScR) Guidelines [14, 15]. Search records were imported into Endnote (Clarivate) [16] for de‐duplication (Figure 1).

A preliminary search was run in Medline (Ovid) and Pubmed. The search was peer‐reviewed using the online PRESS Forum [17], and the Peer Review of Electronic Search Strategies checklist [18]. All databases were searched from January 1, 2004 to September 5, 2025, and included articles published in English only. The search queries were revised and translated for additional electronic databases, including CINAHL (EBSCO), Embase.com (Elsevier), and the Cochrane Library. Google Scholar was searched using Harzing's Publish or Perish [19] tool, and the first 200 records were exported into Endnote for screening. Search strategies are shown in Supporting Information S1.

Three reviewers (KB, SG, IH) independently screened and analyzed studies, including those reporting the incidence of PTH and recurrent PTH in children. The data search found 761 abstracts related to recurrent PTH in children, including CINAHL (81), Cochrane Library (226), Embase (97), Google Scholar (300), and Ovid Medline (57). After exclusion of 83 duplicates, 678 abstracts and titles were reviewed. Inclusion criteria consisted of case reports, case series, retrospective reviews, or randomized controlled trials that included the incidence of recurrent PTH in children. The abstracts were excluded if they did not report recurrent PTH bleed rates or did not primarily include children in the analysis. A total of 12 abstracts [11, 12, 13, 20, 21, 22, 23, 24, 25, 26, 27, 28] met the initial criteria for further review, and, of these, one [28] was excluded due to a limited number of children analyzed. The 11 remaining manuscripts were further reviewed. All 11 manuscripts were case series. This scoping review was guided by the PEO framework, focusing on pediatric patients who experienced recurrent PTH (Population), with an emphasis on repeat episodes of bleeding following an initial PTH (Exposure), and outcomes including incidence, timing, risk factors, and management strategies (Outcome).

Studies were queried for the age of patients, study period, patient sex, total number of tonsillectomy/T&A procedures, number of PTH cases, number of recurrent PTH cases, interventions for initial and recurrent PTH cases, risk factors analyzed for PTH and recurrent PTH cases, i.e., indication for procedure, patient sex, patient age, preoperative bleeding questionnaire or bleeding history, surgical technique, time between initial and recurrent PTH, medications given postoperatively, laboratory data, and presence of clot after the initial PTH. We did not evaluate the methodological quality of the included studies, as this study focused on providing a broad overview of the existing literature.

### 2.2 Statistical Analysis

A meta‐analysis was not feasible due to inconsistencies in reporting across the included studies. Descriptive statistical analyses were utilized, including frequencies of pooled data compiled from the 11 individual studies. Baseline characteristics and clinical factors of the initial PTH and recurrent PTH were summarized as frequency (*n*) and percentages. Risk factors, including exam findings after initial PTH, management of initial PTH, tonsillectomy indication, and type of initial PTH, were compared with Chi‐squared testing to assess for an association with recurrent PTH. Significance was determined at *p* < 0.05. All statistical analyses were performed using Prism GraphPad (741) 9.3.1 (GraphPad Software, San Diego, California USA, www.graphpad.com).

## 3 Results

Variables analyzed in the 11 studies included in this scoping review, including the incidence of initial PTH, the incidence of recurrent PTH, and risk factors for recurrent PTH, are summarized in Table 1. The studies analyzed included 3221 patients who experienced an episode of PTH, with an average age of 7.5 years. Only 6 studies included the total number of tonsillectomies performed during the study period analyzed, and, across these studies, the overall PTH rate was 3.8%. The timing of the initial bleed was variably reported. Where available, the mean was 6.3 days after tonsillectomy (range, 4.6–7.5 days). Table 2 reports the presentation at first PTH, management strategy employed, tonsillectomy indication, tonsillectomy technique, and type of initial PTH (primary or secondary bleed).

**TABLE 1:** Variables analyzed in manuscripts included in scoping review or recurrent PTH.

References	Total tonsillectomy (*n*)	Initial PTH (*n*)	Recurrent PTH (*n*)	Risk for recurrent PTH	Not a risk for recurrent PTH
[20]	11,140	452	32	Age ≥ 12 years BMI ≥ 85 percentile	Abnormal labs, technique, co‐morbidities, bleeding questionnaire, surgery indication
[12]	3866	285	32	Male sex	Blood loss
[21]	5400	234	13		Abnormal labs, NSAID use, technique, severity of initial PTH
[11]		826	22		
[22]		112	10	Age, surgical indication, no OR for initial PTH	
[23]	5015	92	3		
[24]		291	31		Abnormal labs, indication, age, gender
[25]	6810	181	12		
[13]	8660	695	41		No demographic or clinical variables
[26]		424	15		
[27]		72	18	Presence of clot	Age, gender, indication

Abbreviations: BMI, body mass index; OR, operating room; PTH, post‐tonsillectomy hemorrhage.

**TABLE 2:** Variables associated with post‐tonsillectomy hemorrhage (PTH) in the studies analyzed.

Variable analyzed	Total patients *n* (%)	Range	# of studies reporting variable
Total Tonsillectomies	40,891	3866–11,140	6
Initial PTH	3221 (3.8)^a^	55–826	11
Age at first PTH (mean; *n* = 1995)	7.5	6.4–9.1	6
Gender			8
Male	879 (54.4)	29–250	
Female	736 (45.6)	23–202	
Unknown	1606		
POD for first PTH (mean)	6.3	4.6–7.5	8
Presentation at first PTH			6
Active bleeding	300 (15.2)	40–146	
Blood clot	611 (31.0)	28–405	
Normal exam	1058 (53.7)	27–380	
Unknown	1252		
Management of first PTH			9
Observation	1816 (62.2)		
Surgical control	976 (33.4)	0–452	
Bedside intervention	36 (1.2)	0–36	
Discharged	92 (3.2)	0–43	
Unknown	301		
Tonsillectomy indication			6
Recurrent tonsillitis	480 (35.2)	10–169	
Other	884 (64.8)	45–275	
Unknown	1857		
Tonsillectomy technique			5
Intracapsular	73 (6.0)	0–38	
Extracapsular	1154 (94.1)	67–416	
Unknown	1994		
First PTH bleed type			8
Primary bleed	100 (3.8)	0–47	
Secondary bleed	2541 (96.2)	55–826	
Unknown	580		

Abbreviation: POD, postoperative day.

^a^ Percentages based on the 6 studies including the total tonsillectomy number in the analysis.

Across the included studies, 229 patients were described as having recurrent PTH, with numbers ranging from 3 to 41 patients per study. This comprised 7.1% of those who had an initial PTH, and 0.33% who underwent a tonsillectomy where reported. The mean age of patients who rebled was 7.2 years, and the recurrent PTH occurred an average of 9.3 days (range 7.1–10.6 days) after tonsillectomy. Management of recurrent PTH included observation in 83 (38.6%) patients and surgery in 132 (59.7%). Presentation and management of the initial PTH in those with a recurrent PTH are shown in Table 3, in addition to tonsillectomy technique, tonsillectomy indication, and initial PTH type (primary or secondary bleed).

**TABLE 3:** Variables analyzed for those with recurrent post‐tonsillectomy hemorrhage (PTH).

Variable analyzed	Total patients *n* (%)	Range	# of studies reporting variable
Recurrent PTH	229	3–41	11
% out of first PTH	7.1%		
% of total Tonsillectomy	0.33%		
Multiple PTH (> 2)	8	1–3	5
% out of first PTH	0.25%		
Age of patients (mean, *n* = 53)	7.2	6.2–8.3	4
Gender			4
Male	31 (51.7)	6–16	
Female	29 (48.3)	6–16	
Unknown	169		
Postoperative day (mean, days)	9.3	7.1–10.6	6
Management of recurrent PTH			8
Observation	83 (38.6)	0–37	
Surgical	132 (59.7)	9–36	
Interventional radiology	7 (3.1)	1–6	
Unknown	7		
Presentation at initial PTH			5
Normal exam	65 (31.6)	2–41	
Bleeding/clot	57 (46.7)	10–18	
Unknown	107		
Management of initial PTH			9
Surgical	65 (31.6)	3–32	
Observation	141 (68.5)	6–29	
Unknown	23		
Tonsillectomy technique			3
Intracapsular	2 (3.6)	0–2	
Extracapsular	53 (96.4)	10–30	
Unknown	174		
Tonsillectomy indication			2
Recurrent tonsillitis	22 (48.9)	10–12	
Other	23 (51.1)	3–20	
Unknown	184		
Initial PTH bleed type			6
Primary	5 (4.5)	0–3	
Secondary	107 (95.5)	10–30	
Unknown	117		

Potential risk factors for recurrent PTH were inconsistently reported. Presentation at the time of the first PTH, normal exam versus active bleeding/clot in the tonsillar fossa, and the initial management strategy (surgical intervention vs. observation) showed no consistent association with the risk of recurrence. The indications for tonsillectomy and the type of first bleed (primary or secondary) were not associated with recurrence in the available studies when reported (Table 4). Only one study [11] reported administration of TXA to patients reporting with PTH, but the benefits of preventing a recurrent PTH were not analyzed against a control group. This same study evaluated the instrument utilized and found the highest initial PTH rate requiring operative intervention in those undergoing monopolar tonsillectomy, although the rate of further bleeding while patients admitted was similar relative to technique (monopolar cautery, BiZact, Coblation, cold steel, and bipolar cautery). When reported, none of the other studies showed a higher recurrent PTH rate based on instrument used [21, 22].

**TABLE 4:** Analysis of variables associated with recurrent post‐tonsillectomy hemorrhage (PTH).

Variable analyzed	*n*/total	Percentage	*p*
Presentation at First PTH			*p* = 0.10
Normal exam	65/926	7.0	
Bleeding/clot Initial Exam	57/606	9.4	
Management of First PTH			*p* = 0.15
Surgical	65/935	7.0	
Observation	141/1649	8.6	
Tonsillectomy Indication			*p* = 0.06
Recurrent Tonsillitis	22/480	4.5	
Other	23/884	2.6	
Initial PTH Bleed Type			*p* = 0.61
Primary	5/100	5.0	
Secondary	107/2541	4.2	

NSAID use postoperatively was mentioned in 2 manuscripts [20, 21] but was not shown to contribute to an increased rate of recurrent PTH. Laboratory data, including hemoglobin/hematocrit and/or PT/PTT levels, were variably included in the manuscripts reviewed [21, 22, 24, 26]. Although some abnormal values were noted, no consistent association with recurrence was described. Low hemoglobin levels requiring blood transfusions were reported in 12 patients.

## 4 Discussion

Given the large number of tonsillectomies performed yearly in children, otolaryngologists and ED providers are no doubt familiar with the complication of PTH. Management strategies for PTH described in the literature include observation, TXA administration, embolization of feeding vessels, bedside cautery, and surgical cautery. These events can be stressful for the patient, family, and provider, as PTH may require additional trips to the ED, overnight hospital stays, additional operative intervention, blood transfusion, and recurrent bleeding. The overall reported rate of primary and secondary PTH is around 4.2% [2].

Our scoping review sought to describe the incidence, reported risk factors, and management of recurrent PTH in children, and to map existing evidence in this area. Across the 11 included studies, 7.1% of patients presenting with an initial PTH were reported to experience recurrence, which corresponds to approximately 0.33% of all tonsillectomies in cohorts where denominators were provided. Definitions of “recurrent bleeding” and reporting of timing and severity were inconsistent, limiting comparability between studies. No specific risk factors for recurrent PTH were identified, including presentation and management of the initial PTH, tonsillectomy technique, or tonsillectomy indication.

Several of the manuscripts included in this review sought to determine risk factors for recurrent PTH. In their review of 452 PTH cases, McKeon et al. [20] found a recurrent bleed rate of 7.1% (32 patients), and age > 12 years and high body mass index (> 85th percentile) were significantly associated with recurrent PTH. The indication for surgery, surgical technique, intraoperative blood loss, and perioperative medications were not associated with re‐bleeding. Van der Meer et al. [21] evaluated 13/234 PTH cases who had a recurrent bleed, and the authors found no association between abnormal coagulation studies, NSAID use, severity of the first bleed, or surgical technique. Another study [22] of 10/67 pediatric patients with recurrent PTH showed, on regression analysis, that children who were taken to the OR after the initial PTH were less likely to bleed again regardless of cardiac status or indication of surgery. Our cumulative analysis of recurrent PTH did not reveal any clear risk factors. The small number of patients included in some of the analyses reviewed may have influenced their findings, pointing to the need for larger, multi‐center studies on the topic.

Studies included in our analysis evaluated the risk of recurrent PTH in patients admitted overnight after an initial bleeding episode, or if the presence of a clot versus a normal exam of the tonsillar fossa was associated with a higher rate of recurrent PTH [11, 12, 13]. In their analysis of 224 patients admitted with nonactive bleeding, Whelan et al. [12] found the rate of rebleeding, including 26/203 (12.8%) patients managed with observation alone and 3/21 (14.3%) managed with initial surgical intervention, to have no significant difference. The authors commented that observation alone may have comparable safety and efficacy compared to surgical management in those with nonactive bleeding on presentation. Yuen et al. [13] analyzed 337 patients with nonactive bleeding on presentation to the ED, and the authors noted that 38 (11%) required cauterization for rebleeding, and that 32/38 (84%) of these patients bled within 24 h of their admission. Another study [11] noted that 22 (2.9%) patients rebled while being observed with a median time to rebleed of 4.4 h. Our cumulative data showed the first PTH episode occurring 6.3 days postoperatively, and the recurrent PTH 9.3 days. Overall, decisions to admit patients for observation after their initial bleeding episode appear to vary based on presentation and across institutions, but our data noted a 3‐day gap between the bleeding episodes.

Our analysis did not show a difference in recurrent PTH rates based on tonsillectomy indication, tonsillitis versus other indications (i.e., sleep disordered breathing, obstructive sleep apnea), or the tonsillectomy technique (intracapsular versus extracapsular), although only a few of the studies included data on technique and instruments utilized for surgery (only two patients with recurrent PTH underwent the intracapsular technique when reported). The instrument used for tonsillectomy was not noted in most of the studies, and when reported [11, 21, 22], there were multiple instruments used for tonsillectomy even across a single institution, limiting the ability to link the instrument used to recurrent PTH rates. As far as the method of tonsillectomy, a systematic review of randomized controlled trials comparing intracapsular (IT) or partial tonsillectomy technique with total tonsillectomy (TT) technique in pediatric patients showed a significantly lower PTH rate in the IT group when compared to the TT group, in addition to reduced use of analgesics and faster return to a normal diet [29]. The incidence of recurrent PTH was not compared in the systematic review, and our scoping review does not add additional insight into the impact the intracapsular technique might have on recurrent PTH rates given the small numbers.

A couple of the studies [20, 21] included in our analysis evaluated if medications (NSAIDs) given postoperatively affected the rate of recurrent PTH. Neither study showed that NSAID use impacted the recurrent PTH rate. Based on their systematic review and meta‐analysis of the effect and safety of ibuprofen use in pediatric tonsillectomy, Kim et al. [8] did not note a significantly higher PTH rate in patients receiving ibuprofen. Clinically significant bleeding requiring hospital admission or surgical control was not noted in those who received ibuprofen postoperatively in their review, and ibuprofen use reduced the need for analgesic drugs and decreased postoperative nausea and vomiting. In their review of the association between ibuprofen and surgical management of PTH, Mudd et al. [6] did not notice an increased need for surgical management for PTH in patients taking ibuprofen, although hemorrhage severity (based on transfusion requirement) was increased with ibuprofen use. Several patients across the analyses included in our scoping review required blood transfusions for recurrent PTH management. Despite this, with recurrent bleeding rates being so low and limited data on this topic in the studies analyzed, it is difficult to comment on whether patients should be cautioned against continued use of NSAIDs after an initial PTH to decrease the chance of recurrent bleeding.

In only one of the studies [11] included in this analysis was TXA given for the management of the initial PTH, although the risk of recurrent PTH was not specifically analyzed relative to TXA administration. Nebulized TXA has been shown to decrease the need for operative control of bleeding [10, 30]. In their study comparing 100 patients with a PTH who received nebulized TXA, compared to 187 patients who did not, Ojeaga et al. [30] showed higher rates of discharge without surgical intervention and reduced need for surgical intervention in those patients presenting with clot in the tonsillar fossa who received TXA. Despite this, readmission rates for recurrent PTH between the two groups were similar (TXA group 9.0% versus no‐TXA group 7.0%, *p* = 0.52). Intravenous administration of TXA during tonsillectomy was not shown to decrease postoperative PTH risk in a systematic review and meta‐analysis [9], although topical administration of TXA at the time of surgery was shown to reduce the risk of postoperative bleeding. Overall, the impact TXA will have on the rate of recurrent PTH is unclear at this time, but with wider use of TXA for PTH management across the country, more information on the topic of TXA benefits for reducing recurrent PTH will likely be forthcoming.

This scoping review identifies several important gaps in the literature. These include the lack of prospective, multicenter studies designed to identify predictors of recurrent PTH, heterogeneous definitions of a “recurrent bleed,” lack of severity grading and timing, limited and inconsistent reporting of perioperative predictors (surgical technique, medications, and comorbidities), sparse evidence regarding the predictive value of laboratory screening for occult coagulopathies, and lack of standardized management or outcome measures for children with multiple bleeding episodes.

Limitations of this analysis include the small number of studies available and the lack of consistent data points utilized in the studies reviewed. The age ranges included, study duration, risk factors for, and management for recurrent PTH reported were inconsistent between the studies, limiting our ability to completely assess the overall risk and risk factors associated with recurrent bleeding. In addition, the true rate of initial and recurrent PTH episodes may be underappreciated as patients with minor bleeding may not report these events to their otolaryngologist or may seek care at other institutions. Despite this, our scoping review showed a low overall incidence of recurrent PTH. This information should be beneficial when counseling families about bleeding rates after tonsillectomy in children.

## 5 Conclusions

A scoping review of the literature found that among children who experienced PTH, the incidence of recurrent bleeding was approximately 7.1%. In the overall population of children undergoing tonsillectomy, the incidence of recurrent PTH was around 0.33%. Current evidence is fragmented, underpowered, and methodologically heterogeneous. Standardized definitions, consensus reporting guidelines, and prospective multicenter studies are needed to clarify incidence, identify modifiable risk factors, and guide management strategies for children at risk of recurrent hemorrhage.

## Funding

The authors have nothing to report.

## Conflicts of Interest

The authors declare no conflicts of interest.

## Supporting information


**Supporting Information S1:** Recurrent PTH Scoping Review Literature Search.
Box A: Ovid Medline search.
Box B: Embase search.
Box C: Cochrane Library search.
Box D: CINAHL search.
Box E: Google scholars search.

## Data Availability

The data that support the findings of this study are available from the corresponding author upon reasonable request.
