# Supplementary material for: A Scoping Review of Recurrent Post‐Tonsillectomy Hemorrhage in Children
Source: Laryngoscope. 2025 Dec 8;136(5):2026–33. doi: 10.1002/lary.70304 (PMC13067217; doi:10.1002/lary.70304)
Supplement: Supplementary file 1 — Supporting Information S1: Recurrent PTH Scoping Review Literature Search. Box A: Ovid Medline search. Box B: Embase search. Box C: Cochrane Library search. Box D: CINAHL search. Box E: Google scholars search. [file LARY-136-2026-s001.docx]

| 1. Ovid MEDLINE(R) ALL <1946 to September 04, 2025> |
| --- |
| 1 Adolescent/ or Child/ or Child, Preschool/ or Pediatrics/ or (adoles* or child* or juvenile* or paed* or pediatr* or teen* or young person* or young people* or youth).ti,ab. 4388390  2 Adenoidectomy/ or Tonsillectomy/ or (tonsillectomy or adenotonsillectomy or tonsil surger*).ti,ab. 16261  3 Postoperative Hemorrhage/ or (post* adj5 (bleed* or rebleed* or rebled or blood clot* or haemorr* or hemorr*)).ti,ab. 52465  4 ("post* tonsillectomy hemorrhage" or "post* tonsillectomy bleed*").ti,ab. 335  5 Postoperative Care/ or Postoperative Complications/ or Postoperative Period/ or post*.ti,ab. 3617838  6 Hemorrhage/ or (hemorrhag* or haemorr* or clot* or rebleed*).ti,ab. 455368  7 5 and 6 99761  8 3 or 4 or 7 123247  9 Cautery/ or Hemostasis/ or (cautery or haemostasis or hemostasis or intervention).ti,ab. 1012944  10 1 and 2 and 8 and 9 310  11 (repeat* or recurr* or multiple* or subsequent* or "rate of return").ti,ab. 4243649  12 10 and 11 57  13 limit 12 to yr="2024 -Current" 5 |

| 1. Embase |
| --- |
| .......................................................  No. Query Results Results Date  #13. #10 AND #11 AND [2024-2025]/py 14 5 Sep 2025  #12. #10 AND #11 116 5 Sep 2025  #11. repeat*:ti,ab OR recurr*:ti,ab OR multiple*:ti,ab 6,031,186 5 Sep 2025  OR subsequent*:ti,ab OR 'rate of return':ti,ab  #10. #1 AND #2 AND #8 AND #9 525 5 Sep 2025  #9. 'cauterization' OR 'hemostasis' OR cautery:ti,ab 1,601,880 5 Sep 2025  OR haemostasis:ti,ab OR hemostasis:ti,ab OR  intervention:ti,ab  #8. #3 OR #4 OR #7 228,504 5 Sep 2025  #7. #5 AND #6 159,564 5 Sep 2025  #6. hemorrhag*:ti,ab OR haemorr*:ti,ab OR clot*:ti,ab 645,089 5 Sep 2025  OR rebleed*:ti,ab  #5. 'postoperative care' OR 'postoperative 5,304,014 5 Sep 2025  complication' OR 'postoperative period' OR  post*:ti,ab  #4. 'post* tonsillectomy hemorrhage':ti,ab OR 'post 435 5 Sep 2025  tonsillectomy bleed*':ti,ab  #3. 'postoperative hemorrhage' OR ((post* NEAR/5 116,857 5 Sep 2025  (bleed* OR rebleed* OR rebled* OR 'blood clot' OR  haemor* OR hemorr*)):ti,ab)  #2. 'adenoidectomy' OR 'tonsillectomy' OR 27,085 5 Sep 2025  ((tonsillectomy:ti,ab OR adenotonsillectomy:ti,ab  OR tonsil:ti,ab) AND surger*:ti,ab)  #1. 'adolescent'/exp OR 'adolescent' OR 'child'/exp 5,917,741 5 Sep 2025  OR 'child' OR 'pediatrics'/exp OR 'pediatrics' OR  (((adoles*:ti,ab OR child*:ti,ab OR  juvenile*:ti,ab OR paed*:ti,ab OR pediatr*:ti,ab  OR teen*:ti,ab OR young:ti,ab) AND person*:ti,ab  OR young:ti,ab) AND people*:ti,ab) OR youth:ti,ab  ....................................................... |

| 1. Cochrane Library |
| --- |
| Search Name:  Date Run: 05/09/2025 22:31:32  Comment:  ID Search Hits  #1 MeSH descriptor: [Adolescent] explode all trees 138149  #2 MeSH descriptor: [Child] explode all trees 83150  #3 MeSH descriptor: [Pediatrics] explode all trees 1067  #4 (adoles* or child* or juvenile* or paed* or pediatr* or teen* or young person* or young people* or youth):ti,ab,kw 358805  #5 #1 OR #2 OR #3 OR #4 358867  #6 MeSH descriptor: [Adenoidectomy] explode all trees 617  #7 MeSH descriptor: [Tonsillectomy] explode all trees 1299  #8 (tonsillectomy or adenotonsillectomy or tonsil surger*):ti,ab,kw 3562  #9 #6 OR #7 OR #8 3712  #10 MeSH descriptor: [Postoperative Hemorrhage] explode all trees 1789  #11 ((post* adj5 (bleed* or rebleed* or rebled or blood clot* or haemorr* or hemorr*))):ti,ab,kw 0  #12 (post* tonsillectomy hemorrhage):ti,ab,kw 389  #13 (post* tonsillectomy bleed*):ti,ab,kw 498  #14 #10 OR #11 OR #12 OR #13 2324  #15 MeSH descriptor: [Postoperative Care] explode all trees 5282  #16 MeSH descriptor: [Postoperative Complications] explode all trees 56527  #17 MeSH descriptor: [Postoperative Period] explode all trees 7425  #18 #15 OR #16 OR #17 64959  #19 MeSH descriptor: [Hemorrhage] explode all trees 20838  #20 (hemorrhag* or haemorr* or clot* or rebleed*):ti,ab,kw 58510  #21 #19 AND #20 15873  #22 #18 AND #21 2798  #23 #14 OR #22 3324  #24 MeSH descriptor: [Cautery] explode all trees 943  #25 MeSH descriptor: [Hemostasis] explode all trees 5974  #26 (cautery or haemostasis or hemostasis or intervention):ti,ab,kw 635146  #27 #24 OR #25 OR #26 639821  #28 #5 AND #9 AND #23 AND #27 13 results (2024-2025) |

| 1. CINAHL |
| --- |
| S1. ( (MH "Adolescence") OR (MH "Child") OR (MH "Pediatrics") ) OR ( (adoles* OR child* OR juvenile* OR paed* OR pediatr* OR teen* OR "young person*" OR "young people*" OR youth) )  S2. ( (MH "Adenoidectomy") OR (MH "Tonsillectomy") ) OR ( tonsillectom* OR adenotonsillectom* OR "tonsil surger*") )  S3. (MH "Postoperative Hemorrhage") OR "postoperative hemorrhage"  S4. "post* tonsillectomy hemorrhage" OR "post* tonsillectomy bleed*"  S5. ( (MH "Postoperative Care") OR (MH "Postoperative Complications") OR (MH "Postoperative Period") ) OR post*  S6. (MH "Hemorrhage") OR ( (hemorrhag* or haemorr* or clot* or rebleed*) )  S7. S5 AND S6  S8. S3 OR S4 OR S7  S9. (MH "Cautery") OR (MH "Hemostasis") OR cautery or haemostasis or hemostasis or intervention  S10. S1 AND S2 AND S8 AND S9 - 2 results |

| E. Google Scholar  *searched using Harzing’s Publish or Perish tool |
| --- |
| adolescent*\|child* \|paed*\| pediatr*\|teen* tonsillectom*\|adenotonsillectom*\|"tonsil surgery” “postoperative bleeding”\|“postoperative hemorrhage” cautery\|hemostasis\|intervention  100 results (2024-2025) |
